# Observations by a statistical watchdog

**DOI:** 10.48101/ujms.v126.5665

**Published:** 2021-02-08

**Authors:** Adam Taube

**Affiliations:** Department of Statistics, Uppsala University, PO Box 513, SE-75120 Uppsala

**Keywords:** Selection bias, Data torturing, Statistical cleansing, Multiplicity problems, Clinical trials, Survival analysis

## Abstract

During more than five decades, the author has kept a critical eye on how statistical methods are (mis-)used in medical research. Some areas are presented where serious statistical mistakes are prevalent. Two investigations with erroneous conclusions are described in detail. Situations where outside authorities have tried to mute medical researchers are also commented upon. The authors own efforts to improve the use of statistical methods and the current situation with easily accessible statistical program packages are described. Finally, the importance of continued ‘statistical cleansing’ is stressed.

When I started my 60-year random walk in medical statistics, I happened to read a paper in a Swedish medical journal about ‘Science, Statistics and Psychiatry’. It was written in an elegant but arrogant manner by a well-reputed medical doctor and contained some serious misunderstandings. The author seemed to have a high confidence in his own intellectual abilities. Obviously, he was one of many doctors at the time I had heard expressing views like: *I don’t apply any sophistical statistical methods which the reader can’t understand. Instead I use my ordinary common sense. That is quite enough.*

Not seldom, this attitude was combined with a certain ‘statistics hostility’, probably a way of compensating for the lack of statistical knowledge. However, arriving at a wrong conclusion by applying an inadequate statistical method in a wrong way does not tell us anything about the method as such. Further, it does not demonstrate that there is some sort of gap between using ‘ordinary common sense’ and a correct statistical analysis of a scientific problem.

I felt seriously provoked by the article and wrote a paper of my own, presenting some critical arguments. My contribution was formulated in a similar manner as the paper I criticized. I showed the manuscript to the old Uppsala-professor Gunnar Nyström, a retired professor of surgery with an international reputation, a wise man and friend of the family who had recommended me to specialize in medical statistics. He fully agreed on each point in my paper, but was very critical of the arrogant tone in my formulations. Intentionally, I had used the same style as the other author, but he was not satisfied: *It doesn’t matter in which manner the other author has expressed his views, you have to write as a gentleman!*

Of course, I rewrote my manuscript, which was then accepted in the same journal. It was my first publication in a medical journal and my first effort in ‘statistical criticism’.

Unfortunately, I have not always been able to follow the advice ‘to write as a gentleman’. Sometimes, the temptation to formulate stinging remarks has been too strong, and I have felt that in my job as a statistician on a university level, it is important to keep an eye on how standard statistical methods are used and often misused in medical publications. Academic statisticians ought to devote more efforts to this important task. The lack of statistical knowledge or unwillingness to adapt modern statistical methods decreases the scientific quality, and it must be stressed that there are no statistical methods for improving faulty data (‘garbage in–garbage out’). Also, the lack of courage to stand up for scientific values is sometimes disturbing. All these make the role of statistical watchdogs important.

In the following, I will describe how the work on statistical selection mechanisms made me look for such errors in medical research and then widened my interest to all types of statistical mistakes, which I have found in numerous publications. Then, I describe my own efforts to improve the situation. The problems with improper pressure from outside authorities to prevent certain researchers from publishing what they want are taken up, and finally, some recommendations for future activities to improve the situation are given.

## My statistical battlefield

At the end of the fifties, I collaborated with a physician, Willem van der Linden, investigating some data where statistical selection mechanisms were involved. In a medical paper, the famous Prof. Henschen claimed that there was a negative correlation between the occurrence of arteriosclerosis and cancer (1). The basis was to be found in autopsy data. By means of a simple numerical example, Willem and I could demonstrate that this pattern could occur simply due to the different death risks, even when the two diseases were not related at all in the population at risk (PAR), from which the dead patients had come (‘Competition among fatality rates’) (2). It was a simple application of ‘Berkson’s fallacy’, first described in a paper in 1946 (3).

Willem and I wrote a short communication on this statistical trap (4) and also a survey of different kinds of statistical selection mechanisms and selective factors in patient data (5). We then continued to publish a number of papers with statistical criticism until Willem’s death some years ago.

When scrutinizing the statistical analysis in a medical paper, the first step must always be to find out how the data were generated and how they should determine the choice of analysis. This applies both to experimental and non-experimental studies, but it is perhaps more tricky in the latter case, which will be illustrated here.

### Length of hospital stay

Sometimes, studies are concerned with a group of patients with a certain diagnosis without further specifications. If, for example, this group consists of individuals available in a hospital during a certain day, or a certain week, the sample can be biased. The longer a patient is hospitalized, the larger is his/her probability of being included in the study. Thus, the individuals under study will have an overrepresentation of serious cases. This type of bias was already pointed out by Prof. Schwarz in 1957 (6) but one can still come across studies where the authors are not aware of this mechanism or just ignore it. Even many non-medical investigations with this type of bias can be found.

### Only some participants are able to answer

My studies of various selection mechanisms made it easier for me to identify other situations where the data structure could influence the analysis and sometimes even be used as the basis for misleading results, as in an interview study in Africa about women’s age at their first childbirth. The aim was to estimate the mean age for this event. A number of women in the age of 10 to 50 years were asked how old they were when they had their first child. The (arithmetic) mean, directly based on these answers, is of course influenced by the fact that a number of interviewed women haven’t had any children yet. Therefore, the analysis must be adjusted in some way to avoid bias. I have come across several studies, about weaning, first intercourse, etc., where this type of bias was not recognized (or ignored?) by the investigators.

I was confronted with this problem in Ethiopia, where a number of school girls aged between 9 and 17 years were interviewed about their age at menarche, and the aim was to estimate the mean age for this event, an important indicator used in international comparisons. I suggested a new method to adjust for the fact that answers could be expected only from those girls who had started to menstruate (7). Later, the theoretical properties of different approaches to this problem were investigated and compared in detail (8).

### Too many and too small comparison groups

For the study of prognostic factors in, for example, oncology, it happens that a certain group of available patients (by necessity a limited number) are studied with regard to death or relapse with ‘time to event data’. Some already known prognostic variables are studied together with some new (hopefully better) ones. A common approach is to sort the individuals into subgroups with regard to the various background variables. With one dichotomous background variable, there will be two subgroups; with two, there will be four subgroups; with three, eight subgroups; and so on. Then, comparisons between the outcomes in various subgroups are performed.

In a Swedish doctoral work, 49 patients (35 events) were studied with regard to six possible background variables, giving 17 *P*-values. In a certain category of 25 patients, the subgroup comparisons resulted in 27 more *P*-values. Of course, this gave a number of ‘significant differences’ between various groups due to random variation, but the multiplicity problem with all the *P*-values was never addressed, nor the fact that some of the subgroups must have contained very few individuals or were perhaps even empty. A recommended rule of thumb is to have at least 10 events per explanatory variable (9).

In the same work, another analysis on 26 patients was used for 21 significance analyses and 15 Cox regressions. By publishing different analyses of the same data in different journals, the multiplicity problem is sometimes less obvious. It is not surprising that these kinds of studies have revealed extremely few realistic prognostic factors, and it is shocking that such meaningless ‘data-torturing’ can be taken seriously by the medical community and even be published in well-reputed medical journals (10).

### Wrong interpretation of ‘non-significant results’

Even if significance testing is a well-established technique among medical investigators, there is one possible misunderstanding which needs special attention, namely the belief that a statistically non-significant result proves that the null-hypothesis is true. A non-significant result simply shows that it was not possible to reject the null-hypothesis due to random variation, too few observations, or something else.

When I criticized a certain study, the authors answered: *What we find most remarkable is [the fact] that Taube hasn’t understood that the null hypothesis is valid which is demonstrated in Table 4 which leads to the important conclusion that all these sub-sample populations are parts of one [and the same] single ‘mother population’* (11). It is not difficult to find investigations which have been completely jeopardized due to this misunderstanding of what a statistically non-significant difference really means.

### Ignoring the data structure

In some studies, it is obvious that the authors base the statistical analysis on wrong assumptions about the structure of their own data. This happened, for example, in an interview study, where a number of persons, say *n*, were interviewed on three different occasions, and the analysis was performed as if the answers were 3 × *n* statistically independent observations. This gives an exaggerated view of the sample size and neglects the possible variation between the interview occasions.

It would be tempting to continue presenting a list of all the possible mistakes and misinterpretations to be found in some medical papers, but here it is enough to refer to the works by Altman and colleagues. They have initiated vivid academic activities in this field in Great Britain, where critical discussions about statistical methods in medical research seem to be far more established than in Sweden (12).

In my work with statistical criticism of medical papers, I found that the most serious problems were those connected with the data structure. If the structure is given a suitable model in the analysis, it is not a big issue if, say, minor changes of the analysis method give a confidence interval of 90% instead of the stated 95%. However, if the data character or structure is not taken care of in the subsequent analysis, very serious problems can be expected. For all types of structural problems, the results could be dramatically distorted as will be illustrated in the following two examples, where I happened to take part. It is also interesting to see how the persons involved acted during and after the publication of these investigations. Who the researchers were is of no interest here, so no references are given.

## A misinterpreted clinical trial

In the beginning of the nineties, I was contacted by two prominent professors concerning a recent doctoral dissertation from Finland, comparing the mortality among prostate cancer patients treated either with estrogens or orchiectomy. Independently of each other, they both contacted me for a critical inspection of the work.

Thus, I spent part of the summer scrutinizing the dissertation and especially the basic, already published, papers included. The project was presented as a randomized trial, comparing three treatment groups A, B, and C, and there were especially some differences in the outcomes in A and C which aroused suspicions.

I soon discovered that the basis for the dissertation was not one single trial, but two: First, during several years, a randomized trial comparing A and B, and then, for another and different long period of time, a clinical trial comparing B and C. The two trials did not follow identical protocols, the selection criteria were not the same and the randomization procedures were different. Consequently, it was not surprising that the comparisons between the groups A and C gave some ‘mysterious results’ which were quite easy to explain.

In the autumn, I presented my findings in a lecture to a number of specialists from the Nordic countries. The two professors who had initiated my investigation were very enthusiastic. Both stressed how important it was that my findings were published at the soonest.

First, I wrote a research report from the *Department of Statistics*, which I sent to the author and to the professor in Finland, his research supervisor, with polite letters. The latter answered at once, in a hostile way. In summary, he stated that I should refrain from putting my nose into research, which was outside my field of competence.

The next step was to write a ‘Letter to the Editor’ to the actual journal. I thought that this would have more weight, if the two enthusiastic professors also signed it. One of them explained, however, that he didn’t want to disturb his good collaborations with his colleagues in Finland and the other gave the cryptic answer: ‘I remain on my own’. In the end, there was only one, a junior physician, who supported me and signed the letter.

The editor sent my manuscript to the author in Finland who gave a very detailed answer, taking up all my arguments. He concluded that my criticism was fully relevant. After all these, I expected my ‘Letter to the Editor’ to be published quite soon.

Nearly 2 years later, I presented this sequence of events in a lecture at a cancer center in Seattle, and I finished with a slide: *Publication when?* After the lecture, I was approached by a gentleman from the audience, who was very upset. He was a member of the editorial board of the journal. My ‘Letter to the Editor’ appeared in the next issue, and I have reasons to believe that this was due to his intervention.

From all this, we can conclude that courage and support from professors and supervisors is not always to be expected and that it can take a long time until (if ever) a critical remark can reach the readers.

## An inadequate ‘survival analysis’

At an international conference, a Swedish researcher presented a paper where it was claimed that the survival was longer among those prostate cancer patients who had been operated upon than those who had been treated in other ways. The paper was deemed to be very important, and I guess that it had already been accepted in a well-reputed medical journal, without being thoroughly refereed. The paper was also the most important building block in a forthcoming doctoral dissertation in Sweden. I was contacted by the faculty opponent, who was confused by the statistical analysis, but couldn’t exactly point out the possible error.

After some study, we found that the basis for the statistical analysis was all prostate cancer patients in a certain geographic region who had passed away within a specified time period. Retrospectively, the author had collected clinical data concerning these individuals and could assess whether they had been operated upon or not. After this, he made an analysis of the collected data as if they had come from a prospective cohort study. The remarkable circumstance concerning this so-called survival study is of course that the individuals had to die in order to be included. Those patients during the actual period who had survived were not included and the spectacular results concerning longer survival of the operated patients were not valid.

At the public presentation of the dissertation, the opponent raised all these critical comments. There were also several ‘extra opponents’ who had serious objections. In spite of this, the dissertation passed and the paper was published. I wrote a ‘Letter to the Editor’. This was also signed by several important Swedish experts, but obviously, the editor was reluctant to accept our contribution. Therefore, I asked Prof. Marvin Zelen in Boston, who gladly signed together with some other international experts and the letter was finally published in the journal.

One can guess that there were heavy economic interests behind the desire to show that a prostate cancer operation was to be preferred to other alternatives. Each year, the journal had a sort of competition among medical students. If they could identify articles, where some specified important medical achievements were presented, they were automatically included in a lottery and could win scholarships for their future medical studies. The (erroneous) result concerning prostate cancer operations was one of the ‘achievements’, which was considered worth finding. Later, in a sort of yearbook about the most important recent surgical advancements, the erroneous article from Sweden was also included.

## Educational efforts

In 1985, I was asked to give a lecture to the editorial staff of *Läkartidningen* on how to present statistical data in a medical paper. In preparing this, I studied all the issues of the journal during the previous year, and there was indeed much to be commented upon. Instead of a single lecture, I gave a whole study day.

I also wrote a series of critical articles *Studies in medical statistical routines* (13–17). The most important points dealt with the common overuse of ‘significance stars’ and *P*-values and the quite common, questionable tradition of studying one variable at a time in problems where a multivariate approach was more relevant as some detailed cross tabulations or perhaps regression analysis. Probably, this misuse could partly be explained by the fact that the authors were victims of too many elementary medical statistics courses, where they had learned how to handle just one variable, calculating means, standard deviations, and some simple tests between two groups. When time came for multivariate analysis, the course was already over, perhaps after a short presentation of how to calculate a correlation coefficient.

I found it somewhat of a paradox that medical doctors, who are forced to a multivariate approach when considering all aspects concerning each single patient, so often studied just one variable at a time when presenting a medical investigation. It should be noted though that in the eighties, multivariate statistical methods were not so easily accessible as they are today.

## Pressure from outside

My activities in statistical criticism had some unexpected consequences. I was contacted by medical doctors/researchers who had observed heavy misuse of statistics in their professional environment, but explained that they could not act freely. If they talked about their critical views or published anything about them, they would be victims of various sanctions from above. Therefore, they asked *me* to write about *their* problems, which I also did in a number of cases.

Another situation I have experienced several times in connection with my statistics courses for doctoral students in the medical faculty: After the lecture, somebody waits until all the others have left and then asks me about a statistical problem in his/her doctoral project. But I have to promise not to say anything to anybody, since the medical supervisor does not tolerate consultations with outside statisticians.

An example of the efforts to mute independent doctors/researchers: In a large register study of cardiac deaths, a ranking list of the Swedish clinics was presented by the *National Board of Health and Welfare, Socialstyrelsen*. In this, one particular clinic was pointed out as especially inefficient. The Board was straightforward in presenting this ‘finding’ to the general public. Some tabloid papers even warned potential patients not to attend the clinic.

Two senior doctors at the clinic performed a thorough investigation of all the cases reported from the clinic to the register and found a number of inadequacies and mistakes, which fully explained the disastrous statistical results. However, this was never heard of at the official press conference with representatives both from the local staff and *Socialstyrelsen.*

Before this event, the local doctors had been carefully instructed in a written memorandum from the chief administrator of the county council how to behave: *Irrespective of whether the Medical Board is right or wrong, we have to keep a collegial, humble attitude, especially if we present any criticism about them. Be responsible, admit that we take it very seriously (The National Board of Health and Welfare is not something you can simply neglect!) and that we will change our routines accordingly, as soon as possible.*

In a paper about statistical difficulties and mistakes in extensive register studies that I submitted soon after the above event, I included a quotation from the current memorandum, but it was never included (18). It was the only time when an editor has deleted something from my text *after* I had said OK to the last galley proof.

In spite of this negative experience, I must admit that on the whole, I have had great freedom to present and publish my critical views. It is obvious that in a number of situations, the researchers are not free from censorship. Therefore, it is highly desirable that people outside the medical establishment can point out (statistical) errors in medical research. I have been able to be critical and publish my arguments because I have not been affiliated with any medical department, especially so during my last 10 years before retirement, when I was employed by the *Swedish Cancer Society*, where my criticism was very much encouraged. I was surprised and disappointed, however, that I got so little support from my statistical colleagues in the academic world.

## Today and tomorrow

The negative attitude toward statistical methods seems to have changed. The situation today, 60 years after my first attempts at statistical criticism, is indeed quite different. A number of new statistical methods have been developed and refined approaches in the design of clinical trials are now applied. Multivariate methods, like the Cox Regression, are widely accepted by medical researchers.

This development has been possible due to easily available statistical packages. The arsenal of statistical techniques has exploded. Unfortunately, this generates a high risk for the misuse of statistical methods. It is not difficult to find studies where the choice of statistical approach seems to be based more on the availability of certain programs than the knowledge of statistical methods and principles. Also, during the last decades, a number of national registers have been created, which sometimes are used for statistical analyses made by people with scanty knowledge of statistical principles (19).

Unfortunately, some of the misbehaviors that I have come across can be expected to occur even in the future, such as the lack of courage among some supervisors/professors, the lack of support from above, and the pressure from certain authorities to prevent medical researchers from publishing what they want.

## Cleansing can never cease

When I published a critical comment some years ago about a questionable statistical analysis in a medical paper, the authors answered that *Professor Taube has misinterpreted our paper. It was not intended to be a scientific article, only a report to the Socialstyrelsen.* This indicates a disturbing and unacceptable dualism in the quality standards of data presentations and conclusions.

This attitude is even more directly expressed in a warning on a report from a nutrition institute: *The studies between these covers do not have as their object a high scientific level of precision, performed as they are, during a very short term and on small samples. They are meant to provide rough indications and trends of informative value for decision makers and others, who work in a practical local context*. Such efforts to defend questionable investigations are of course illogical and necessitate continued statistical cleansing. Personally, I have great difficulty in accepting that it is more important that data be reliable in a scientific paper than when they are used as a basis for real-life decisions. I can’t help thinking it ought to be the other way around.

It is no doubt unethical to perform an erroneous statistical analysis, deliberately or not, but I always hope that mostly such mistakes are not intentional. Even so, they can lead to wrong conclusions and inefficient, inadequate, or harmful treatments. Therefore, it will always be necessary to keep a critical eye on medical publications. It is an important task for statisticians on an academic level, and their contributions to the critical discussions in various journals would also be recognized as academic merits.

Sometimes, people have asked me: *Don’t you get many enemies when you write a critical article?*

My answer is: *Yes indeed! But for every enemy, I usually get at least two new good friends*.
